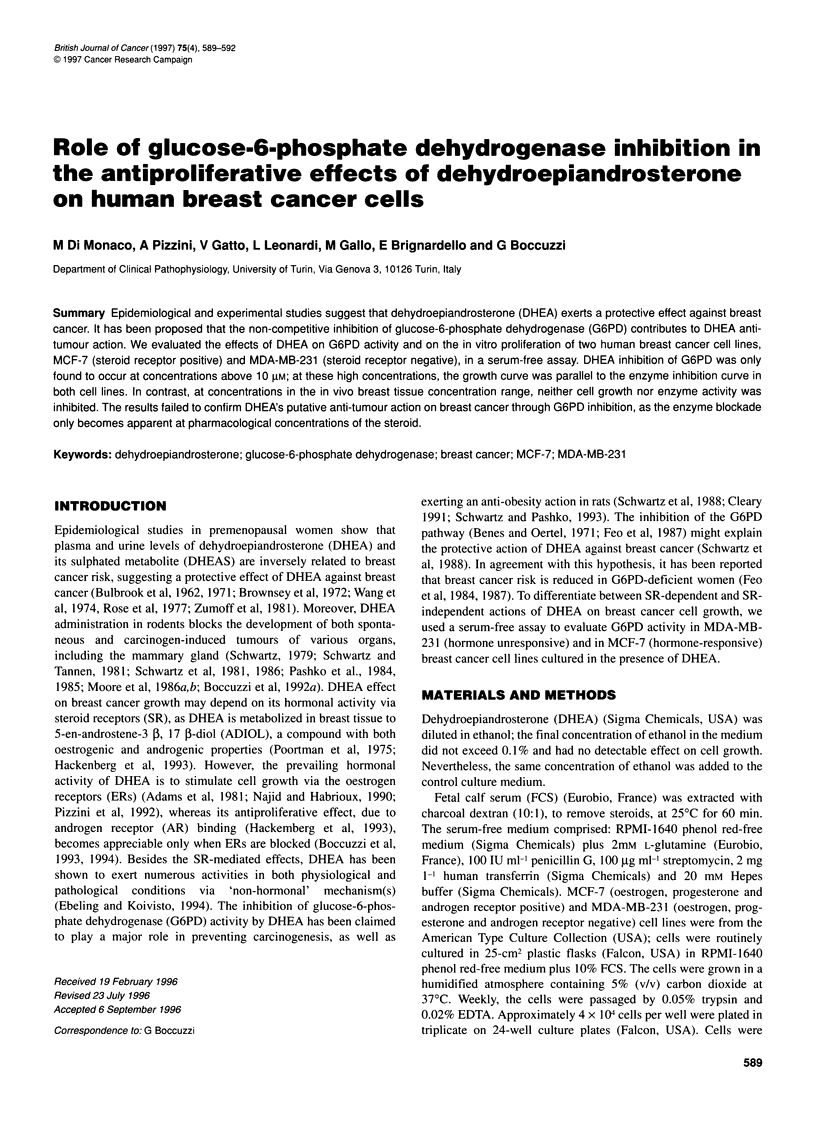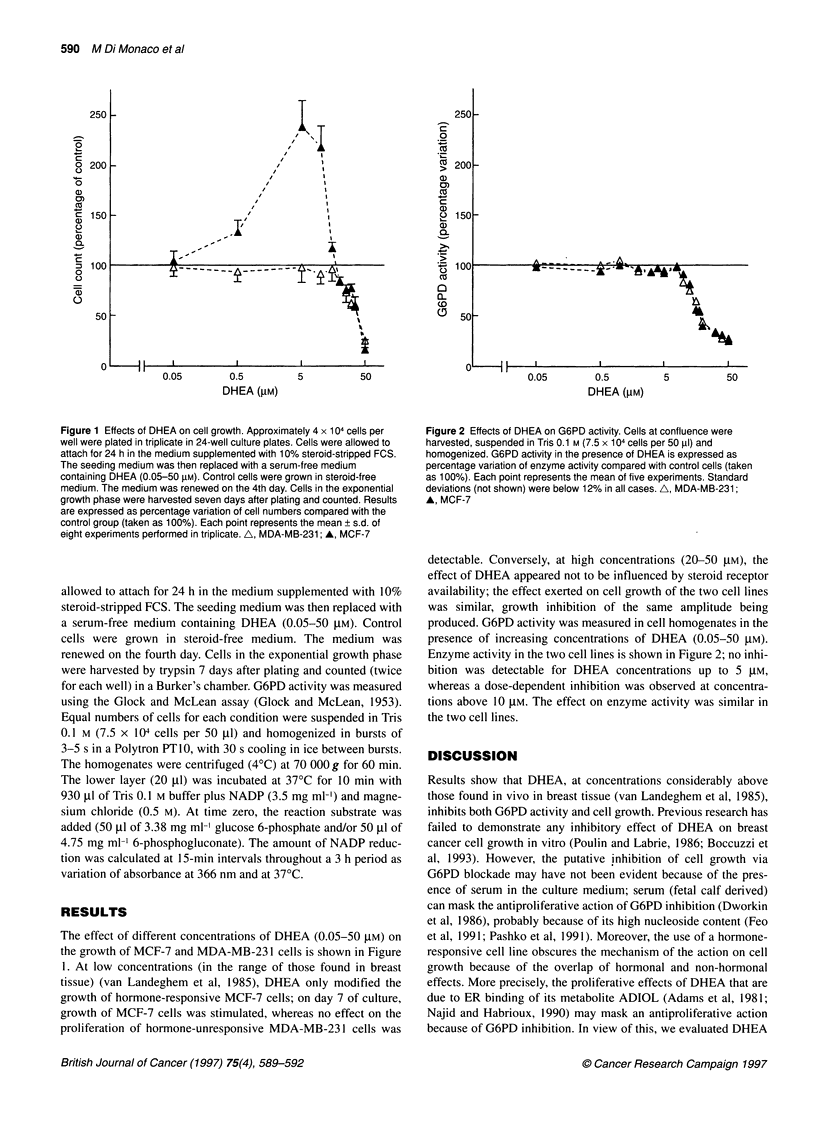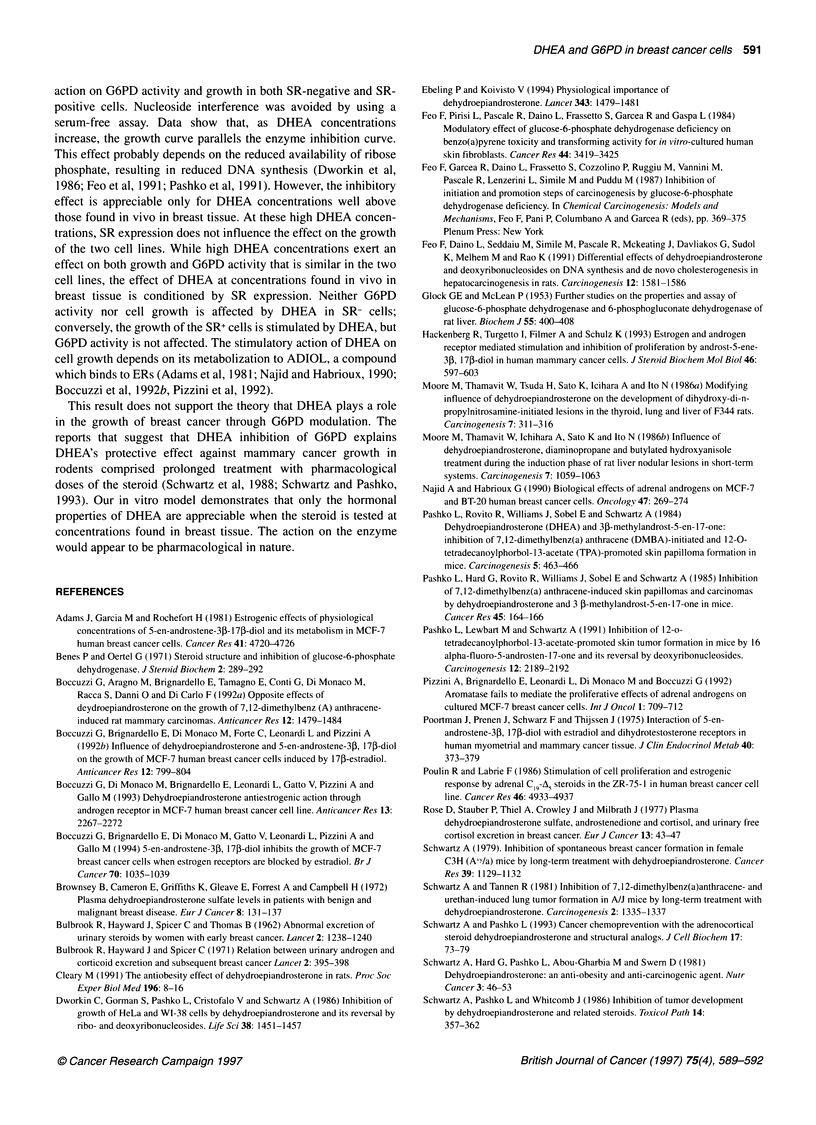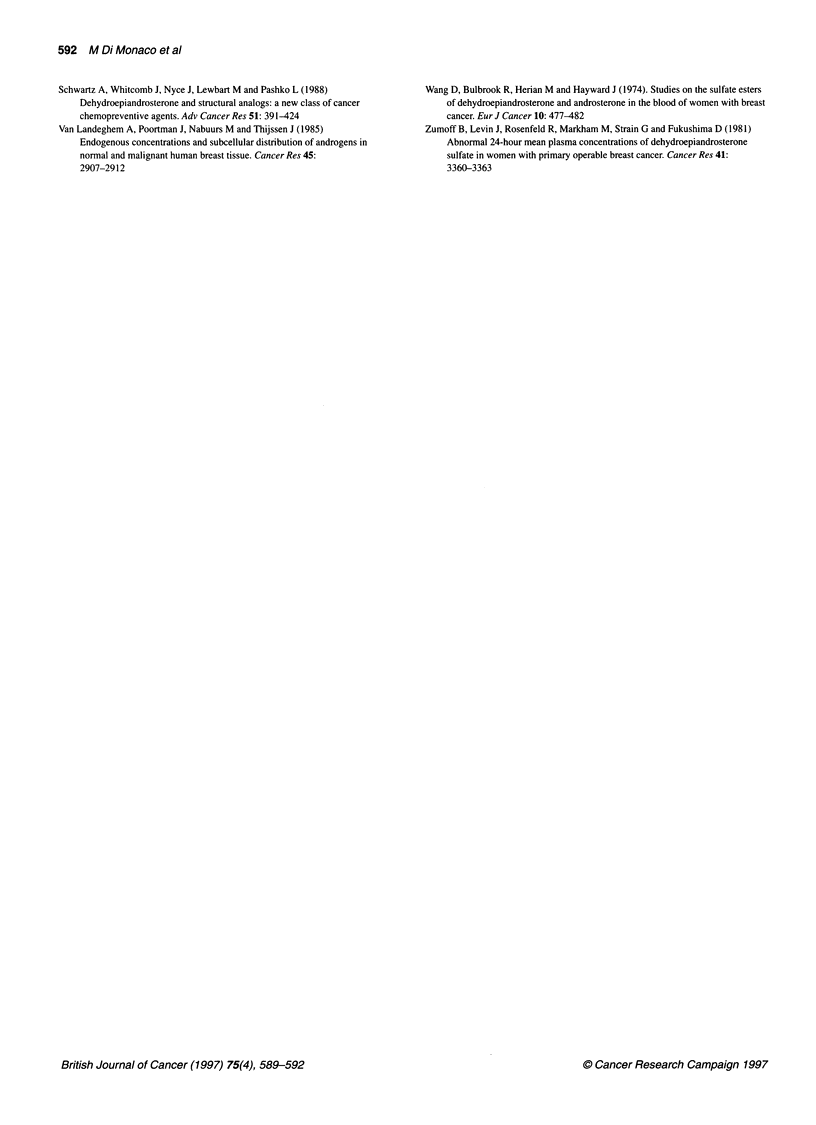# Role of glucose-6-phosphate dehydrogenase inhibition in the antiproliferative effects of dehydroepiandrosterone on human breast cancer cells.

**DOI:** 10.1038/bjc.1997.102

**Published:** 1997

**Authors:** M. Di Monaco, A. Pizzini, V. Gatto, L. Leonardi, M. Gallo, E. Brignardello, G. Boccuzzi

**Affiliations:** Department of Clinical Pathophysiology, University of Turin, Italy.

## Abstract

Epidemiological and experimental studies suggest that dehydroepiandrosterone (DHEA) exerts a protective effect against breast cancer. It has been proposed that the non-competitive inhibition of glucose-6-phosphate dehydrogenase (G6PD) contributes to DHEA antitumor action. We evaluated the effects of DHEA on G6PD activity and on the in vitro proliferation of two human breast cancer cell lines, MCF-7 (steroid receptor positive) and MDA-MB-231 (steroid receptor negative), in a serum-free assay. DHEA inhibition of G6PD was only found to occur at concentrations above 10 microM; at these high concentrations, the growth curve was parallel to the enzyme inhibition curve in both cell lines. In contrast, at concentrations in the in vivo breast tissue concentration range, neither cell growth nor enzyme activity was inhibited. The results failed to confirm DHEA's putative anti-tumor action on breast cancer through G6PD inhibition, as the enzyme blockade only becomes apparent at pharmacological concentrations of the steroid.


British Journal of Cancer (1997) 75(4), 589–592
© 1997 Cancer Research Campaign

### Role of glucose-6-phosphate dehydrogenase inhibition in the antiproliferative effects of dehydroepiandrosterone on human breast cancer cells

**M Di Monaco, A Pizzini, V Gatto, L Leonardi, M Gallo, E Brignardello and G Boccuzzi**

Department of Clinical Pathophysiology, University of Turin, Via Genova 3, 10126 Turin, Italy

**Summary** Epidemiological and experimental studies suggest that dehydroepiandrosterone (DHEA) exerts a protective effect against breast cancer. It has been proposed that the non-competitive inhibition of glucose-6-phosphate dehydrogenase (G6PD) contributes to DHEA anti-tumour action. We evaluated the effects of DHEA on G6PD activity and on the in vitro proliferation of two human breast cancer cell lines, MCF-7 (steroid receptor positive) and MDA-MB-231 (steroid receptor negative), in a serum-free assay. DHEA inhibition of G6PD was only found to occur at concentrations above 10 μM; at these high concentrations, the growth curve was parallel to the enzyme inhibition curve in both cell lines. In contrast, at concentrations in the in vivo breast tissue concentration range, neither cell growth nor enzyme activity was inhibited. The results failed to confirm DHEA's putative anti-tumour action on breast cancer through G6PD inhibition, as the enzyme blockade only becomes apparent at pharmacological concentrations of the steroid.

**Keywords:** dehydroepiandrosterone; glucose-6-phosphate dehydrogenase; breast cancer; MCF-7; MDA-MB-231

### INTRODUCTION

Epidemiological studies in premenopausal women show that plasma and urine levels of dehydroepiandrosterone (DHEA) and its sulphated metabolite (DHEAS) are inversely related to breast cancer risk, suggesting a protective effect of DHEA against breast cancer (Bulbrook et al, 1962, 1971; Brownsey et al, 1972; Wang et al, 1974, Rose et al, 1977; Zumoff et al, 1981). Moreover, DHEA administration in rodents blocks the development of both spontaneous and carcinogen-induced tumours of various organs, including the mammary gland (Schwartz, 1979; Schwartz and Tannen, 1981; Schwartz et al, 1981, 1986; Pashko et al., 1984, 1985; Moore et al, 1986*a,b*; Boccuzzi et al, 1992*a*). DHEA effect on breast cancer growth may depend on its hormonal activity via steroid receptors (SR), as DHEA is metabolized in breast tissue to 5-en-androstene-3 β, 17 β-diol (ADIOL), a compound with both oestrogenic and androgenic properties (Poortman et al, 1975; Hackenberg et al, 1993). However, the prevailing hormonal activity of DHEA is to stimulate cell growth via the oestrogen receptors (ERs) (Adams et al, 1981; Najid and Habrioux, 1990; Pizzini et al, 1992), whereas its antiproliferative effect, due to androgen receptor (AR) binding (Hackemberg et al, 1993), becomes appreciable only when ERs are blocked (Boccuzzi et al, 1993, 1994). Besides the SR-mediated effects, DHEA has been shown to exert numerous activities in both physiological and pathological conditions via 'non-hormonal' mechanism(s) (Ebeling and Koivisto, 1994). The inhibition of glucose-6-phosphate dehydrogenase (G6PD) activity by DHEA has been claimed to play a major role in preventing carcinogenesis, as well as exerting an anti-obesity action in rats (Schwartz et al, 1988; Cleary 1991; Schwartz and Pashko, 1993). The inhibition of the G6PD pathway (Benes and Oertel, 1971; Feo et al, 1987) might explain the protective action of DHEA against breast cancer (Schwartz et al, 1988). In agreement with this hypothesis, it has been reported that breast cancer risk is reduced in G6PD-deficient women (Feo et al, 1984, 1987). To differentiate between SR-dependent and SR-independent actions of DHEA on breast cancer cell growth, we used a serum-free assay to evaluate G6PD activity in MDA-MB-231 (hormone unresponsive) and in MCF-7 (hormone-responsive) breast cancer cell lines cultured in the presence of DHEA.

*Received 19 February 1996*
*Revised 23 July 1996*
*Accepted 6 September 1996*

*Correspondence to:* G Boccuzzi

### MATERIALS AND METHODS

Dehydroepiandrosterone (DHEA) (Sigma Chemicals, USA) was diluted in ethanol; the final concentration of ethanol in the medium did not exceed 0.1% and had no detectable effect on cell growth. Nevertheless, the same concentration of ethanol was added to the control culture medium.

Fetal calf serum (FCS) (Eurobio, France) was extracted with charcoal dextran (10:1), to remove steroids, at 25°C for 60 min. The serum-free medium comprised: RPMI-1640 phenol red-free medium (Sigma Chemicals) plus 2mM L-glutamine (Eurobio, France), 100 IU $ml^{-1}$ penicillin G, 100 μg $ml^{-1}$ streptomycin, 2 mg $l^{-1}$ human transferrin (Sigma Chemicals) and 20 mM Hepes buffer (Sigma Chemicals). MCF-7 (oestrogen, progesterone and androgen receptor positive) and MDA-MB-231 (oestrogen, progesterone and androgen receptor negative) cell lines were from the American Type Culture Collection (USA); cells were routinely cultured in 25-$cm^2$ plastic flasks (Falcon, USA) in RPMI-1640 phenol red-free medium plus 10% FCS. The cells were grown in a humidified atmosphere containing 5% (v/v) carbon dioxide at 37°C. Weekly, the cells were passaged by 0.05% trypsin and 0.02% EDTA. Approximately $4 \times 10^4$ cells per well were plated in triplicate on 24-well culture plates (Falcon, USA). Cells were

Figure 1 Effects of DHEA on cell growth. Approximately 4 x $10^4$ cells per well were plated in triplicate in 24-well culture plates. Cells were allowed to attach for 24 h in the medium supplemented with 10% steroid-stripped FCS. The seeding medium was then replaced with a serum-free medium containing DHEA (0.05–50 μM). Control cells were grown in steroid-free medium. The medium was renewed on the 4th day. Cells in the exponential growth phase were harvested seven days after plating and counted. Results are expressed as percentage variation of cell numbers compared with the control group (taken as 100%). Each point represents the mean ± s.d. of eight experiments performed in triplicate. △, MDA-MB-231; ▲, MCF-7

Figure 2 Effects of DHEA on G6PD activity. Cells at confluence were harvested, suspended in Tris 0.1 M (7.5 x $10^4$ cells per 50 μl) and homogenized. G6PD activity in the presence of DHEA is expressed as percentage variation of enzyme activity compared with control cells (taken as 100%). Each point represents the mean of five experiments. Standard deviations (not shown) were below 12% in all cases. △, MDA-MB-231; ▲, MCF-7

allowed to attach for 24 h in the medium supplemented with 10% steroid-stripped FCS. The seeding medium was then replaced with a serum-free medium containing DHEA (0.05–50 μM). Control cells were grown in steroid-free medium. The medium was renewed on the fourth day. Cells in the exponential growth phase were harvested by trypsin 7 days after plating and counted (twice for each well) in a Burker's chamber. G6PD activity was measured using the Glock and McLean assay (Glock and McLean, 1953). Equal numbers of cells for each condition were suspended in Tris 0.1 M (7.5 × $10^4$ cells per 50 μl) and homogenized in bursts of 3–5 s in a Polytron PT10, with 30 s cooling in ice between bursts. The homogenates were centrifuged (4°C) at 70 000 *g* for 60 min. The lower layer (20 μl) was incubated at 37°C for 10 min with 930 μl of Tris 0.1 M buffer plus NADP (3.5 mg $ml^{-1}$) and magnesium chloride (0.5 M). At time zero, the reaction substrate was added (50 μl of 3.38 mg $ml^{-1}$ glucose 6-phosphate and/or 50 μl of 4.75 mg $ml^{-1}$ 6-phosphogluconate). The amount of NADP reduction was calculated at 15-min intervals throughout a 3 h period as variation of absorbance at 366 nm and at 37°C.

### RESULTS

The effect of different concentrations of DHEA (0.05–50 μM) on the growth of MCF-7 and MDA-MB-231 cells is shown in Figure 1. At low concentrations (in the range of those found in breast tissue) (van Landeghem et al, 1985), DHEA only modified the growth of hormone-responsive MCF-7 cells; on day 7 of culture, growth of MCF-7 cells was stimulated, whereas no effect on the proliferation of hormone-unresponsive MDA-MB-231 cells was detectable. Conversely, at high concentrations (20–50 μM), the effect of DHEA appeared not to be influenced by steroid receptor availability; the effect exerted on cell growth of the two cell lines was similar, growth inhibition of the same amplitude being produced. G6PD activity was measured in cell homogenates in the presence of increasing concentrations of DHEA (0.05–50 μM). Enzyme activity in the two cell lines is shown in Figure 2; no inhibition was detectable for DHEA concentrations up to 5 μM, whereas a dose-dependent inhibition was observed at concentrations above 10 μM. The effect on enzyme activity was similar in the two cell lines.

### DISCUSSION

Results show that DHEA, at concentrations considerably above those found in vivo in breast tissue (van Landeghem et al, 1985), inhibits both G6PD activity and cell growth. Previous research has failed to demonstrate any inhibitory effect of DHEA on breast cancer cell growth in vitro (Poulin and Labrie, 1986; Boccuzzi et al, 1993). However, the putative inhibition of cell growth via G6PD blockade may have not been evident because of the presence of serum in the culture medium; serum (fetal calf derived) can mask the antiproliferative action of G6PD inhibition (Dworkin et al, 1986), probably because of its high nucleoside content (Feo et al, 1991; Pashko et al, 1991). Moreover, the use of a hormone-responsive cell line obscures the mechanism of the action on cell growth because of the overlap of hormonal and non-hormonal effects. More precisely, the proliferative effects of DHEA that are due to ER binding of its metabolite ADIOL (Adams et al, 1981; Najid and Habrioux, 1990) may mask an antiproliferative action because of G6PD inhibition. In view of this, we evaluated DHEA

© Cancer Research Campaign 1997

action on G6PD activity and growth in both SR-negative and SR-positive cells. Nucleoside interference was avoided by using a serum-free assay. Data show that, as DHEA concentrations increase, the growth curve parallels the enzyme inhibition curve. This effect probably depends on the reduced availability of ribose phosphate, resulting in reduced DNA synthesis (Dworkin et al, 1986; Feo et al, 1991; Pashko et al, 1991). However, the inhibitory effect is appreciable only for DHEA concentrations well above those found in vivo in breast tissue. At these high DHEA concentrations, SR expression does not influence the effect on the growth of the two cell lines. While high DHEA concentrations exert an effect on both growth and G6PD activity that is similar in the two cell lines, the effect of DHEA at concentrations found in vivo in breast tissue is conditioned by SR expression. Neither G6PD activity nor cell growth is affected by DHEA in $SR^-$ cells; conversely, the growth of the $SR^+$ cells is stimulated by DHEA, but G6PD activity is not affected. The stimulatory action of DHEA on cell growth depends on its metabolization to ADIOL, a compound which binds to ERs (Adams et al, 1981; Najid and Habrioux, 1990; Boccuzzi et al, 1992*b*, Pizzini et al, 1992).

This result does not support the theory that DHEA plays a role in the growth of breast cancer through G6PD modulation. The reports that suggest that DHEA inhibition of G6PD explains DHEA's protective effect against mammary cancer growth in rodents comprised prolonged treatment with pharmacological doses of the steroid (Schwartz et al, 1988; Schwartz and Pashko, 1993). Our in vitro model demonstrates that only the hormonal properties of DHEA are appreciable when the steroid is tested at concentrations found in breast tissue. The action on the enzyme would appear to be pharmacological in nature.

### REFERENCES

Adams J, Garcia M and Rochefort H (1981) Estrogenic effects of physiological concentrations of 5-en-androstene-3β-17β-diol and its metabolism in MCF-7 human breast cancer cells. *Cancer Res* **41**: 4720–4726

Benes P and Oertel G (1971) Steroid structure and inhibition of glucose-6-phosphate dehydrogenase. *J Steroid Biochem* **2**: 289–292

Boccuzzi G, Aragno M, Brignardello E, Tamagno E, Conti G, Di Monaco M, Racca S, Danni O and Di Carlo F (1992*a*) Opposite effects of deydroepiandrosterone on the growth of 7,12-dimethylbenz (A) anthracene-induced rat mammary carcinomas. *Anticancer Res* **12**: 1479–1484

Boccuzzi G, Brignardello E, Di Monaco M, Forte C, Leonardi L and Pizzini A (1992*b*) Influence of dehydroepiandrosterone and 5-en-androstene-3β, 17β-diol on the growth of MCF-7 human breast cancer cells induced by 17β-estradiol. *Anticancer Res* **12**: 799–804

Boccuzzi G, Di Monaco M, Brignardello E, Leonardi L, Gatto V, Pizzini A and Gallo M (1993) Dehydroepiandrosterone antiestrogenic action through androgen receptor in MCF-7 human breast cancer cell line. *Anticancer Res* **13**: 2267–2272

Boccuzzi G, Brignardello E, Di Monaco M, Gatto V, Leonardi L, Pizzini A and Gallo M (1994) 5-en-androstene-3β, 17β-diol inhibits the growth of MCF-7 breast cancer cells when estrogen receptors are blocked by estradiol. *Br J Cancer* **70**: 1035–1039

Brownsey B, Cameron E, Griffiths K, Gleave E, Forrest A and Campbell H (1972) Plasma dehydroepiandrosterone sulfate levels in patients with benign and malignant breast disease. *Eur J Cancer* **8**: 131–137

Bulbrook R, Hayward J, Spicer C and Thomas B (1962) Abnormal excretion of urinary steroids by women with early breast cancer. *Lancet* **2**: 1238–1240

Bulbrook R, Hayward J and Spicer C (1971) Relation between urinary androgen and corticoid excretion and subsequent breast cancer *Lancet* **2**: 395–398

Cleary M (1991) The antiobesity effect of dehydroepiandrosterone in rats. *Proc Soc Exper Biol Med* **196**: 8–16

Dworkin C, Gorman S, Pashko L, Cristofalo V and Schwartz A (1986) Inhibition of growth of HeLa and WI-38 cells by dehydroepiandrosterone and its reversal by ribo- and deoxyribonucleosides. *Life Sci* **38**: 1451–1457

Ebeling P and Koivisto V (1994) Physiological importance of dehydroepiandrosterone. *Lancet* **343**: 1479–1481

Feo F, Pirisi L, Pascale R, Daino L, Frassetto S, Garcea R and Gaspa L (1984) Modulatory effect of glucose-6-phosphate dehydrogenase deficiency on benzo(a)pyrene toxicity and transforming activity for *in vitro*-cultured human skin fibroblasts. *Cancer Res* **44**: 3419–3425

Feo F, Garcea R, Daino L, Frassetto S, Cozzolino P, Ruggiu M, Vannini M, Pascale R, Lenzerini L, Simile M and Puddu M (1987) Inhibition of initiation and promotion steps of carcinogenesis by glucose-6-phosphate dehydrogenase deficiency. In *Chemical Carcinogenesis: Models and Mechanisms*, Feo F, Pani P, Columbano A and Garcea R (eds), pp. 369–375 Plenum Press: New York

Feo F, Daino L, Seddaiu M, Simile M, Pascale R, Mckeating J, Davliakos G, Sudol K, Melhem M and Rao K (1991) Differential effects of dehydroepiandrosterone and deoxyribonucleosides on DNA synthesis and de novo cholesterogenesis in hepatocarcinogenesis in rats. *Carcinogenesis* **12**: 1581–1586

Glock GE and McLean P (1953) Further studies on the properties and assay of glucose-6-phosphate dehydrogenase and 6-phosphogluconate dehydrogenase of rat liver. *Biochem J* **55**: 400–408

Hackenberg R, Turgetto I, Filmer A and Schulz K (1993) Estrogen and androgen receptor mediated stimulation and inhibition of proliferation by androst-5-ene-3β, 17β-diol in human mammary cancer cells. *J Steroid Biochem Mol Biol* **46**: 597–603

Moore M, Thamavit W, Tsuda H, Sato K, Icihara A and Ito N (1986*a*) Modifying influence of dehydroepiandrosterone on the development of dihydroxy-di-n-propylnitrosamine-initiated lesions in the thyroid, lung and liver of F344 rats. *Carcinogenesis* **7**: 311–316

Moore M, Thamavit W, Ichihara A, Sato K and Ito N (1986*b*) Influence of dehydroepiandrosterone, diaminopropane and butylated hydroxyanisole treatment during the induction phase of rat liver nodular lesions in short-term systems. *Carcinogenesis* **7**: 1059–1063

Najid A and Habrioux G (1990) Biological effects of adrenal androgens on MCF-7 and BT-20 human breast cancer cells. *Oncology* **47**: 269–274

Pashko L, Rovito R, Williams J, Sobel E and Schwartz A (1984) Dehydroepiandrosterone (DHEA) and 3β-methylandrost-5-en-17-one: inhibition of 7,12-dimethylbenz(a) anthracene (DMBA)-initiated and 12-O-tetradecanoylphorbol-13-acetate (TPA)-promoted skin papilloma formation in mice. *Carcinogenesis* **5**: 463–466

Pashko L, Hard G, Rovito R, Williams J, Sobel E and Schwartz A (1985) Inhibition of 7,12-dimethylbenz(a) anthracene-induced skin papillomas and carcinomas by dehydroepiandrosterone and 3 β-methylandrost-5-en-17-one in mice. *Cancer Res* **45**: 164–166

Pashko L, Lewbart M and Schwartz A (1991) Inhibition of 12-o-tetradecanoylphorbol-13-acetate-promoted skin tumor formation in mice by 16 alpha-fluoro-5-androsten-17-one and its reversal by deoxyribonucleosides. *Carcinogenesis* **12**: 2189–2192

Pizzini A, Brignardello E, Leonardi L, Di Monaco M and Boccuzzi G (1992) Aromatase fails to mediate the proliferative effects of adrenal androgens on cultured MCF-7 breast cancer cells. *Int J Oncol* **1**: 709–712

Poortman J, Prenen J, Schwarz F and Thijssen J (1975) Interaction of 5-en-androstene-3β, 17β-diol with estradiol and dihydrotestosterone receptors in human myometrial and mammary cancer tissue. *J Clin Endocrinol Metab* **40**: 373–379

Poulin R and Labrie F (1986) Stimulation of cell proliferation and estrogenic response by adrenal $C_{19}$-$\Delta_5$ steroids in the ZR-75-1 in human breast cancer cell line. *Cancer Res* **46**: 4933–4937

Rose D, Stauber P, Thiel A, Crowley J and Milbrath J (1977) Plasma dehydroepiandrosterone sulfate, androstenedione and cortisol, and urinary free cortisol excretion in breast cancer. *Eur J Cancer* **13**: 43–47

Schwartz A (1979). Inhibition of spontaneous breast cancer formation in female C3H ($A^{vy}$/a) mice by long-term treatment with dehydroepiandrosterone. *Cancer Res* **39**: 1129–1132

Schwartz A and Tannen R (1981) Inhibition of 7,12-dimethylbenz(a)anthracene- and urethan-induced lung tumor formation in A/J mice by long-term treatment with dehydroepiandrosterone. *Carcinogenesis* **2**: 1335–1337

Schwartz A and Pashko L (1993) Cancer chemoprevention with the adrenocortical steroid dehydroepiandrosterone and structural analogs. *J Cell Biochem* **17**: 73–79

Schwartz A, Hard G, Pashko L, Abou-Gharbia M and Swern D (1981) Dehydroepiandrosterone: an anti-obesity and anti-carcinogenic agent. *Nutr Cancer* **3**: 46–53

Schwartz A, Pashko L and Whitcomb J (1986) Inhibition of tumor development by dehydroepiandrosterone and related steroids. *Toxicol Path* **14**: 357–362

© Cancer Research Campaign 1997

Schwartz A, Whitcomb J, Nyce J, Lewbart M and Pashko L (1988) Dehydroepiandrosterone and structural analogs: a new class of cancer chemopreventive agents. *Adv Cancer Res* **51**: 391–424

Van Landeghem A, Poortman J, Nabuurs M and Thijssen J (1985) Endogenous concentrations and subcellular distribution of androgens in normal and malignant human breast tissue. *Cancer Res* **45**: 2907–2912

Wang D, Bulbrook R, Herian M and Hayward J (1974). Studies on the sulfate esters of dehydroepiandrosterone and androsterone in the blood of women with breast cancer. *Eur J Cancer* **10**: 477–482

Zumoff B, Levin J, Rosenfeld R, Markham M, Strain G and Fukushima D (1981) Abnormal 24-hour mean plasma concentrations of dehydroepiandrosterone sulfate in women with primary operable breast cancer. *Cancer Res* **41**: 3360–3363

© Cancer Research Campaign 1997